# *In vitro* dynamics of rumen microbiota and fermentation profiles with Antler growth of Sika deer

**DOI:** 10.1128/spectrum.02829-24

**Published:** 2025-01-28

**Authors:** Songze Li, Shaoying Wang, Yuhang Zhu, Ruina Mu, Tao Wang, Yuguo Zhen, Huazhe Si, Rui Du, Zhipeng Li

**Affiliations:** 1 College of Animal Science and Technology, Jilin Agricultural University 85112 , Changchun, China; 2 Key Lab of Animal Production, Product Quality and Security, Ministry of Education, Jilin Agricultural University, Changchun, China; 3 College of Chinese Medicine Materials, Jilin Agricultural University 85112 , Changchun, China; 4 Joint International Research Laboratory of Modern Agricultural Technology, Ministry of Education, Jilin Agricultural University, Changchun 130118, China; 5 Jilin Provincial Engineering Research Center for Efficient Breeding and Product Development of Sika Deer, Jilin Agricultural University 85112 , Changchun, China; Jilin University, Changchun, China

**Keywords:** *in vitro *fermentation, antler growth, butyrate, rumen microbiota

## Abstract

The rumen microbiota plays a vital role in the nutrient metabolism affecting the growth of velvet antler. However, the fermentation patterns and dynamics of the rumen microbiota across growth stages of velvet antler remain largely unexplored. Here, we employed an *in vitro* fermentation approach to assess fermentation parameters and microbial composition in the rumen liquid of sika deer during the early growth (EG), metaphase growth (MG), and fast growth (FG) phases . Our findings indicated that the levels of short-chain fatty acids (SCFAs), ammonia nitrogen, and gas production increased over fermentation time in all three groups. The concentrations of total SCFAs, ammonia nitrogen, and gas production, along with the proportions of butyrate, isobutyrate, and isovalerate, were markedly higher in the MG and FG groups compared with the EG group. Principal coordinate analysis highlighted significant variations in microbial communities among the EG, MG, and FG groups during the fermentation process. The abundances of *Stomatobaculum* and *Blautia* across the three groups increased over fermentation time, whereas *Bacteroides*, *Lawsonibacter*, *Sporobacter*, *Papillibacter*, *Butyricicoccus*, and *Succiniclasticum* exhibited higher abundances in MG or FG groups than in the EG group after 24 hours of fermentation. Co-occurrence network analysis uncovered positive correlations between butyrate levels and butyrate-producing bacteria (*Stomatobaculum*, *Butyrivibrio*) in the MG and FG groups. Additionally, there were positive correlations between proteolytic bacteria (*Clostridium* and *Roseburia*) and branched-chain volatile fatty acids in the FG group. These findings shed light on the fermentation patterns and microbial dynamics within the rumen of sika deer during different growth periods of velvet antler.

**IMPORTANCE:**

Velvet antlers are distinctive and rapidly growing organs that hold significant value in traditional medicine. Through *in vitro* analysis, our study characterized the dynamics of microbiota and metabolites within the rumen liquid fermentation of sika deer throughout the different antler growth phase. We identified distinct microbial communities at various fermentation time points and observed shifts in fermentation patterns that paralleled antler development. These findings suggest a potentially pivotal role for these microbial dynamics in facilitating the growth process of velvet antlers.

## INTRODUCTION

Cervidae are globally farmed for their venison and valuable by-products including skins and velvet antlers, which are utilized in traditional medicine (1). The most prevalent species in farming include sika deer (*Cervus nippon*), red deer (*Cervus elaphus*), and reindeer (*Rangifer tarandus*). Velvet antlers, regarded as secondary sexual characteristics, are primarily composed of cartilage and osteoblasts, which are unique as the only fully regenerative tissue in mammals, exhibiting rapid growth from extra vertebrae (2). The rapid growth of velvet antlers is facilitated by thick arteries that ensure a steady supply of nutrients to the circulatory system (3), underscoring a potential connection between nutrient metabolism and velvet antler development (4). Indeed, previous research has indicated that velvet antler growth has a high demand for nutrients, particular energy and protein (5). Consequently, it is essential to understand the relationship between nutrient metabolism and velvet antler growth for enhancing productivity.

Like other ruminants, the rumen is the primary site for nutrient degradation in cervids, where microbial fermentation generates vital metabolites, such as short-chain fatty acids (SCFAs), which are pivotal to the host’s physiology and energy metabolism (6, 7). In our previous research, we conducted a comparative analysis of the rumen microbiota and fermentation parameters among sika deer, red deer, and their hybrids, revealing that the relative abundances of *Prevotella* and Succinivibrionaceae, as well as the concentration of butyrate, were linked to the weight of velvet antlers (7, 8). Recent research on the rumen microbiota of sika deer during the early growth (EG), metaphase growth (MG), and fast growth (FG) phases further suggests that the production of SCFAs (particularly propionate and butyrate) and the metabolism of specific amino acids (arginine, proline, alanine, aspartate, and glutamate) are integral to velvet antler growth (9). Butyrate, a key metabolite from microbial fermentation, is recognized for its influence on bone growth by activating the Wnt signaling pathway in osteoblasts (10) and also serves as a potent inhibitor of osteoclasts, reducing both their formation and resorptive activity, thus playing a role in bone metabolism regulation (11). Moreover, studies have indicated that an increased abundance of *Fibrobacter* and elevated SCFAs levels in the rumen of sika deer are associated with enhanced velvet antler production (12). These findings indicate a link between velvet antler growth and the rumen composition, as well as the metabolites they produce.

It’s widely recognized that SCFAs and ammonia nitrogen generated in the rumen are absorbed and utilized by the rumen epithelial tissues through passive diffusion and nitrogen recycling (13). The ability and efficiency of SCFA absorption by the rumen epithelium directly impact the pH levels and SCFA production within the rumen, which subsequently influences the host’s energy metabolism (14). These insights imply that *in vivo* measurements in the rumen might not fully account for microbiota-induced SCFA production. Therefore, it is essential to examine the microbial dynamics responsible for SCFA production in the rumen of sika deer across different stages of velvet antler growth. *In vitro* fermentation technology can mimic the rumen fermentation kinetics, offering a straightforward and potent method to assess the metabolic capacity of the rumen microbiota (15). This approach circumvents the influence of host interference factors, such as rumen passage rate and absorption, and has been extensively used to elucidate nutrient metabolism in ruminants (16). Thus, in this study, we explored the metabolite profiles and microbial community composition in the rumen of sika deer at distinct time points by employing an *in vitro* fermentation approach and full-length 16S rRNA gene sequencing.

## RESULTS

### The rumen liquid fermentation parameters *in vitro* over time

**Fig 1:**
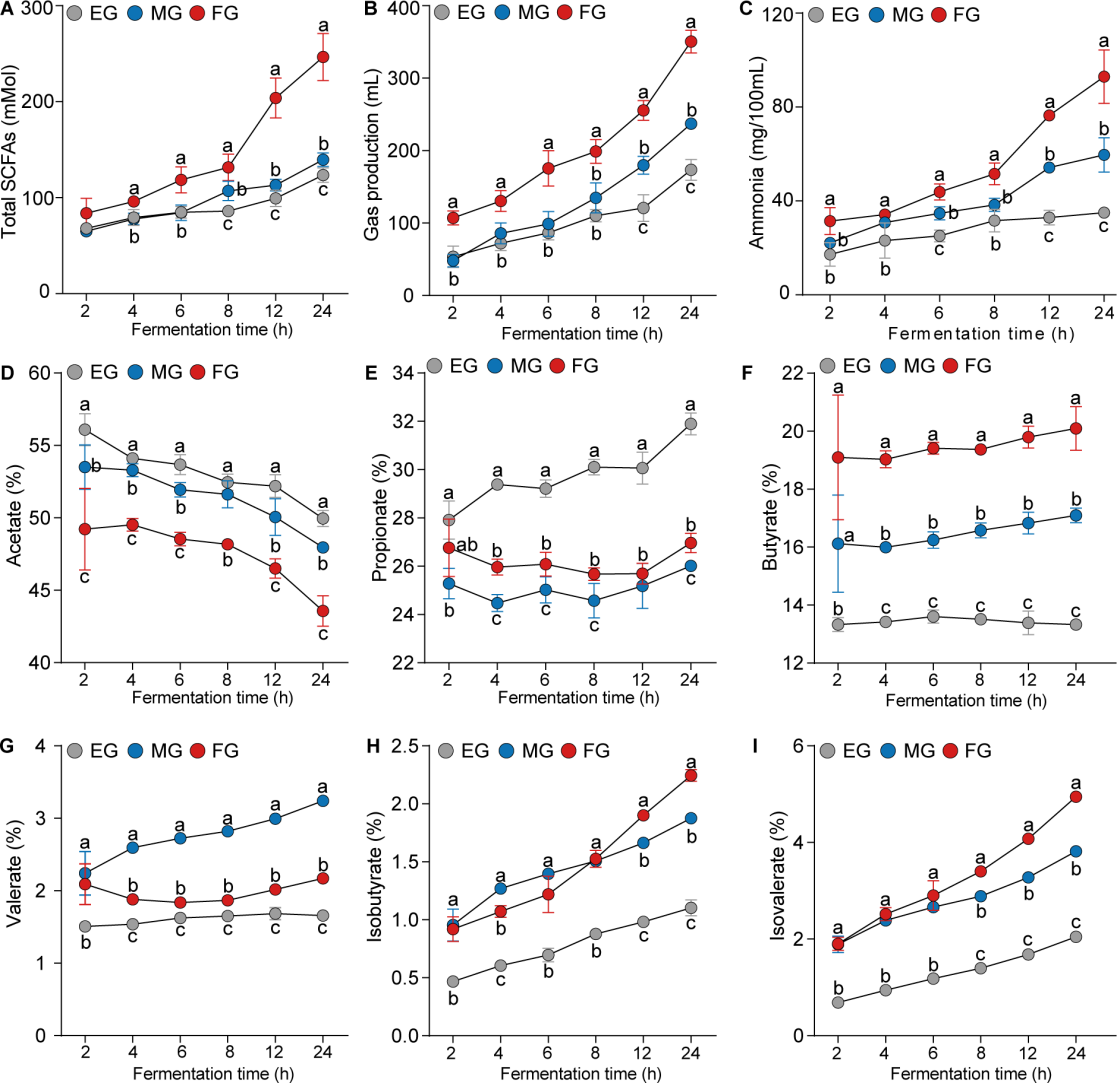
Comparison of rumen liquid fermentation parameters *in vitro*. The concentrations of total SCFAs (**A**), gas production (**B**), and concentrations of ammonia nitrogen (**C**) in rumen fermentation liquid. Molar proportions of acetate (**D**), propionate (**E**), butyrate (**F**), valerate (**G**), isobutyrate (**H**), and isovalerate (**I**) in rumen fermentation liquid. a, b, and c indicate *P* < 0.05 among the three groups at the same time.

The results showed that the concentrations of total SCFAs including acetate, propionate, butyrate, isobutyrate, valerate, and isovalerate increased with fermentation time across the EG, MG, and FG groups (*P* < 0.05; Fig. 1A ; Fig. S1A through F). Conversely, the pH declined (Fig. S1G). The molar proportion of acetate decreased over the course of *in vitro* fermentation for all groups (Fig. 1D), whereas the molar proportion of propionate increased in the EG group (Fig. 1E). Meanwhile, the molar proportion of butyrate increased in both the MG and FG groups as fermentation progressed (Fig. 1F).

We then compared the fermentation parameters across the EG, MG, and FG groups. Our analysis revealed that the FG group exhibited higher concentrations of total SCFAs, ammonia nitrogen, and gas production compared with the EG and MG groups throughout the *in vitro* fermentation process, with significant differences observed at 12 and 24 hours (*P* < 0.05; Fig. 1A through C). Specifically, the concentration of total SCFAs and ammonia nitrogen and the gas production in the FG group were approximately double those of the EG group at 24 hours. However, the molar proportions of acetate and propionate showed a decreasing and increasing trend over fermentation time in all three groups, respectively. The molar proportions of acetate and propionate in the MG and FG groups were significantly lower than those in the EG group (*P* < 0.05; Fig. 1D through E). Additionally, the molar proportions of butyrate, valerate, isobutyrate, and isovalerate in the FG and MG groups were significantly higher than those in the EG group (*P* < 0.05; Fig. 1F through I).

### Characteristics of the microbial community over *in vitro* fermentation time

**Fig 2:**
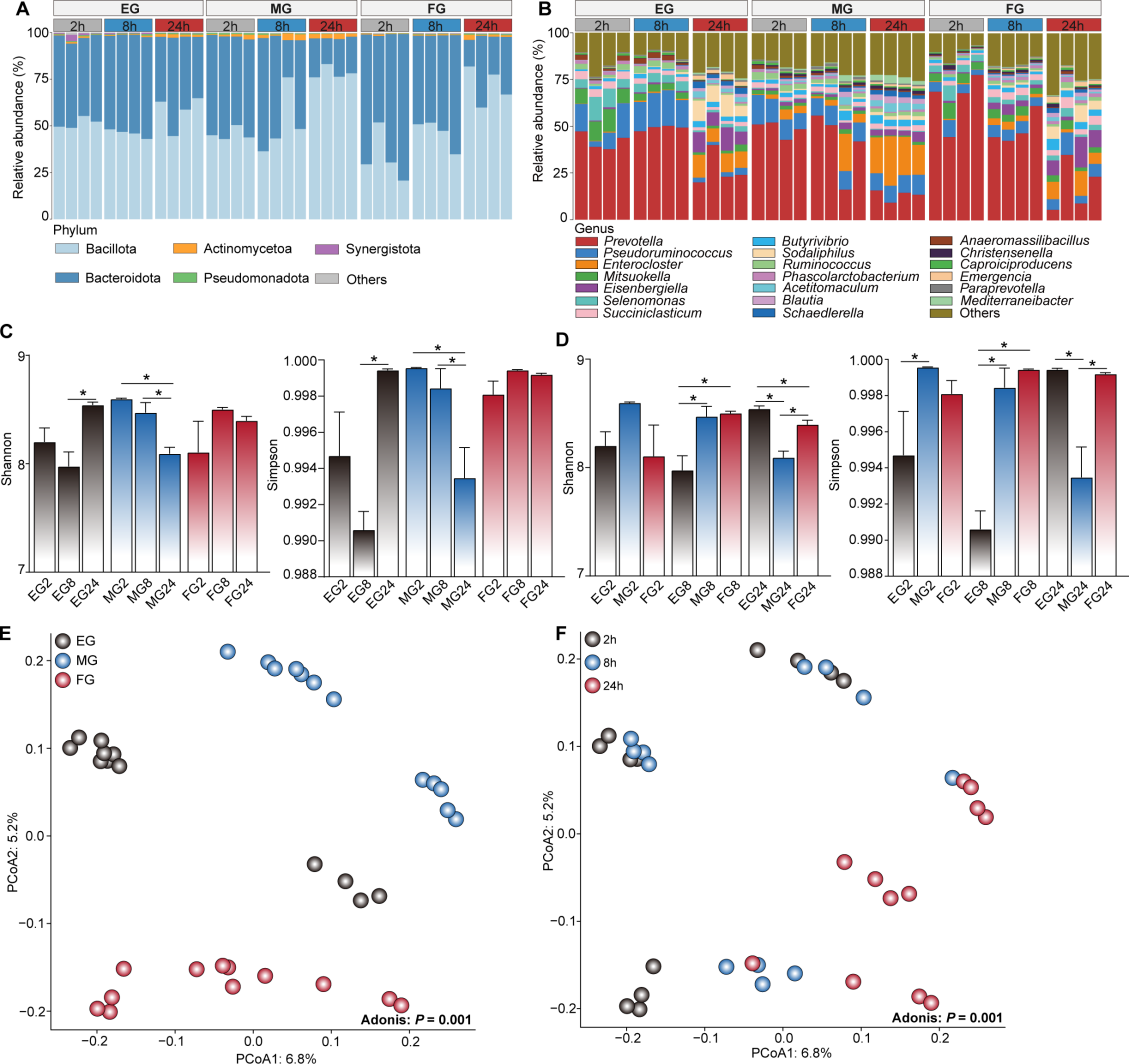
Microbial community composition and structure in rumen fermentation liquid over time. Microbial composition at the phylum (**A**) and genus (**B**) levels. Comparison of alpha diversity indices among the EG, MG, and FG groups with fermentation time (**C**) and among EG, MG, and FG groups at the same fermentation time (**D**). PCoA results showing the change of microbial community in the rumen liquid among the EG (EG2, EG8, and EG24), MG (MG2, MG8, and MG24), and FG (FG2, FG8, and FG24) groups (**E**) and among the different fermentation time (2 hours = EG2, MG2, and FG2; 8 hours = EG8, MG8, and FG8; and 24 hours = EG24, MG24, and FG24) (**F**) based on Unweighted UniFrac distance at OTU level. * indicates *P* < 0.05.

Given the notable changes in fermentation parameters at 2, 8, and 24 hours, we then examined the microbial community at these time points for the EG (EG2, EG8, and EG24), MG (MG2, MG8, and MG24), and FG (FG2, FG8, and FG24) groups using the full length of the 16S rRNA gene sequencing. A total of 461,040 high-quality reads were generated from the EG, MG, and FG groups, with an average of 12,806 reads per sample (ranging from 7,095 to 20,569), which were clustered into 2,453 operational taxonomic units (OTUs). Further classification identified 19 phyla, with Bacillota and Bacteroidota predominating across the three groups (Fig. 2A). A total of 388 genera were further identified, with *Prevotella* (13.36%–64.75 %), *Pseudoruminococcus* (2.58%–16.94 %), *Enterocloster* (0.29%–20.51 %), *Mitsuokella* (0.93%–9.49 %), and *Eisenbergiella* (0.59%–9.32 %) being the most abundant (Fig. 2B; Table S2 ). Analysis of the alpha diversity indices within each EG, MG, and FG group indicated that the Shannon and Simpson indices significantly decreased in the MG group as fermentation time progressed (*P* < 0.05; Fig. 2C). Additionally, when comparing the alpha diversity indices at the same time points among the groups, the Shannon and Simpson indices at 8 hours in the MG and FG groups were higher than those in the EG group, while at 24 hours, the indices in the EG and FG groups were higher than those in the MG group (*P* < 0.05; Fig. 2D). Principal coordinate analysis (PCoA) results demonstrated significant differences in the microbial community composition among the EG, MG, and FG groups (*P* = 0.001; Fig. 2E), as well as among different time points (*P* = 0.001; Fig. 2F), based on Unweighted UniFrac distance metrics.

### Microbial composition shifts over fermentation time

**Fig 3:**
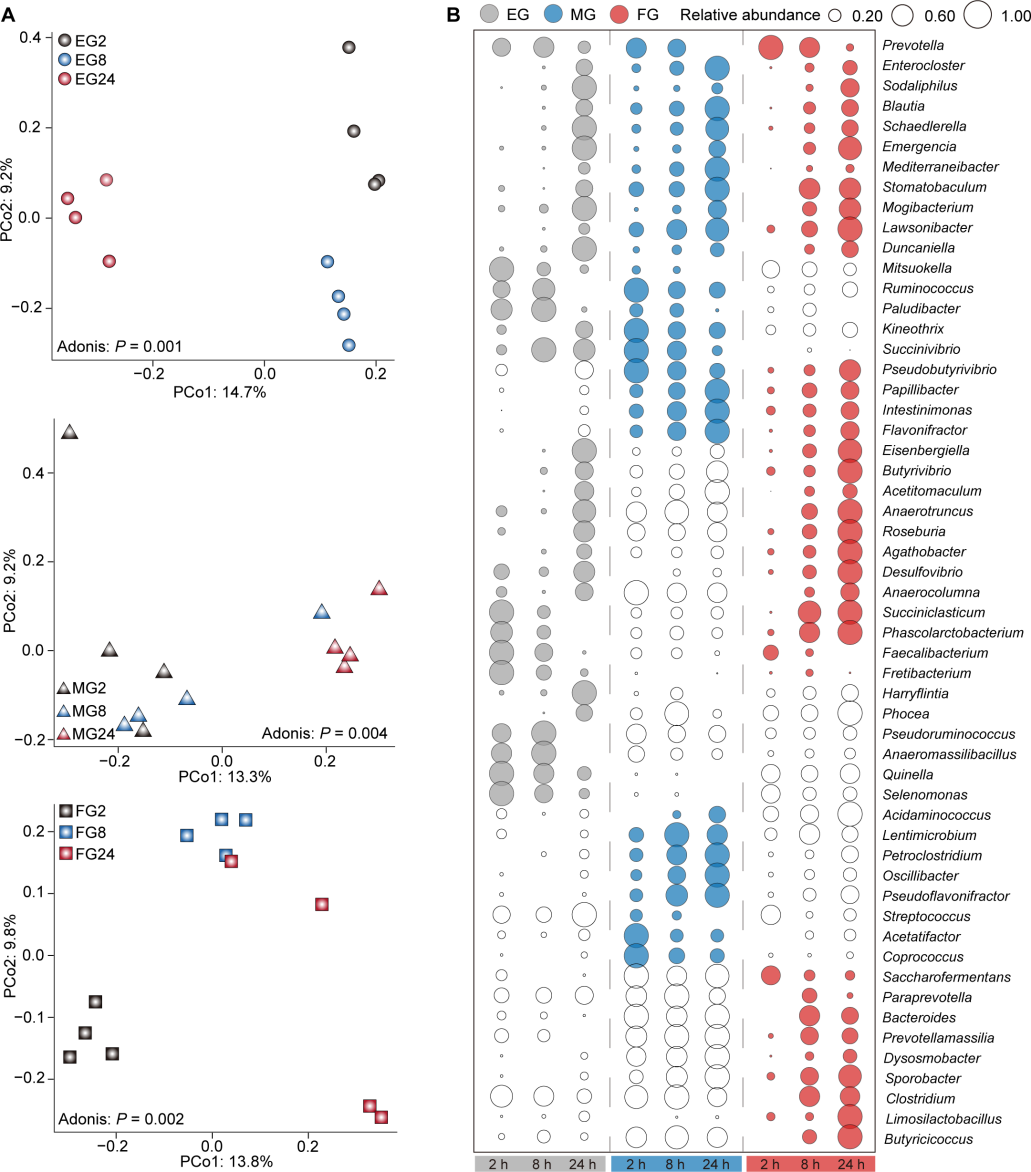
The variations of rumen microbiota with fermentation time. (**A**) PCoA results revealing changes among the EG, MG, and FG groups over fermentation time based on Unweighted UniFrac distance at OTU level. (**B**) Bubble graphs showing significantly changed genera among the EG, MG, and FG groups with fermentation time. While bubbles indicate no significant difference among the three time points.

We initially compared the microbial composition at 2, 8, and 24 hours within the EG, MG, and FG groups separately. Significant differences in the microbial community were observed for the EG (*P* = 0.001), MG (*P* = 0.004), and FG (*P* = 0.002; Fig. 3A) groups, respectively. We then identified the microbial composition changes across the three time points for each group (*P* < 0.05; Fig. 3B; Table S3 through S5). The relative abundances of *Enterocloster* and *Blautia* increased significantly in the EG, MG, and FG groups over fermentation time, while the proportion of *Prevotella* notably decreased. For both the MG and FG groups, the relative abundances of *Papillibacter*, *Intestinimonas*, and *Flavonifractor* significantly rose from 2 hours to 24 hours. In comparison to those at 2 hours, the relative abundances of *Eisenbergiella*, *Butyrivibrio*, *Acetitomaculum*, *Anaerotruncus*, *Roseburia*, *Agathobacter*, *Desulfovibrio*, and *Anaerocolumna* significantly increased at 24 hours in both the EG and FG groups. The relative abundances of *Selenomonas* and *Pseudoruminococcus* in the EG group significantly diminished over fermentation time. The relative abundances of *Petroclostridium*, *Oscillibacter*, and *Pseudoflavonifractor*, in the MG group, and those of *Sporobacter*, *Butyricicoccus*, and *Clostridium*, in the FG groups, increased over fermentation time.

**Fig 4:**
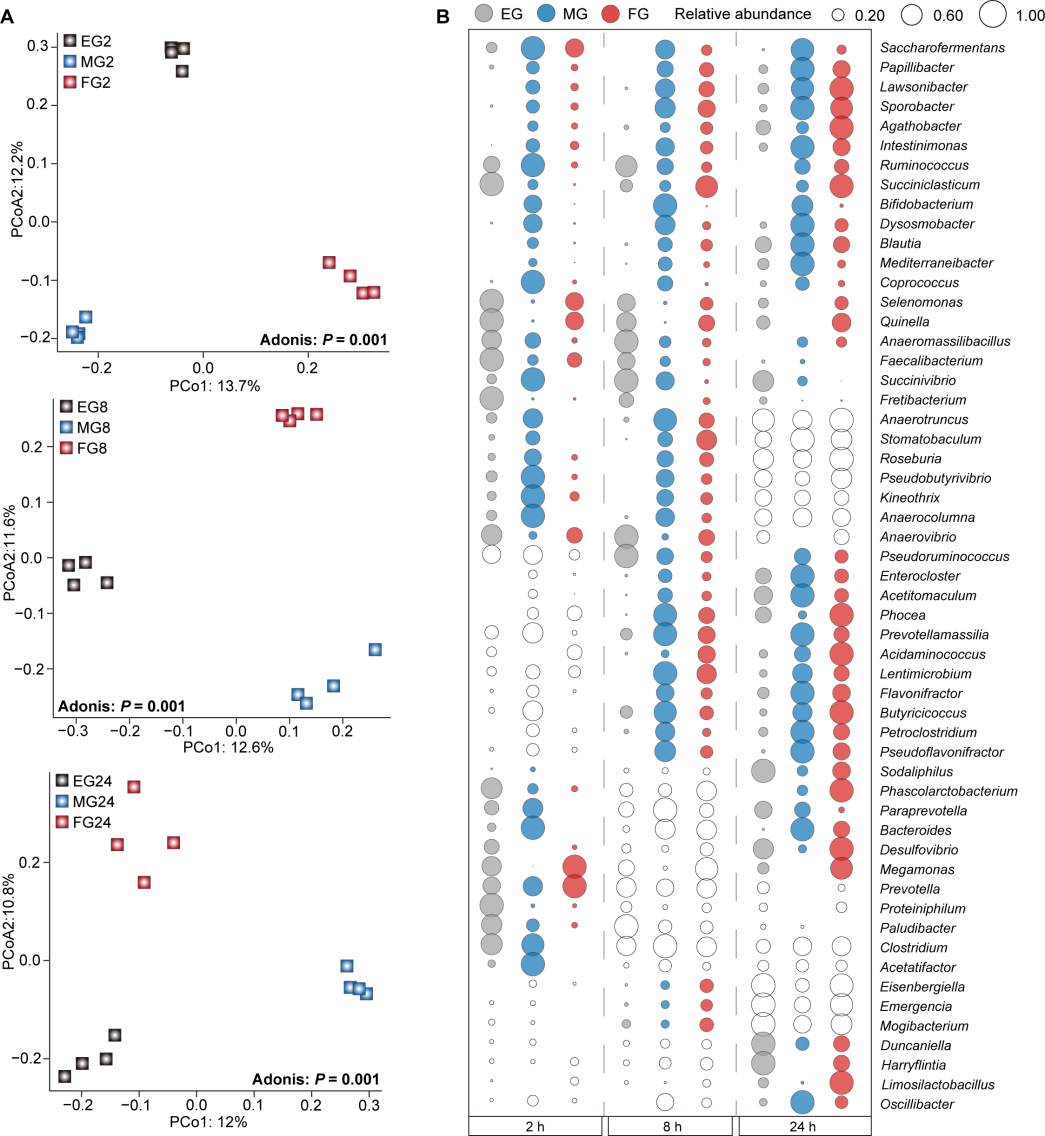
The variations of rumen microbiota at the same fermentation time among the EG, MG, and FG groups. (**A**) PCoA results revealing the changes in rumen microbiota at 2 hours, 8 hours, and 24 hours for the EG, MG, and FG groups, respectively, based on Unweighted UniFrac distance at OTU level. (**B**) Bubble graphs showing significantly changed genera among the EG, MG, and FG groups at 2 hours, 8 hours, and 24 hours, respectively. Gray, blue, and red colors represent the EG, MG, and FG groups, respectively. White bubbles indicate no significant difference among the three groups.

Furthermore, differences in the microbial community were observed among the EG, MG, and FG groups at 2 hours, 8 hours, and 24 hours, respectively (*P* = 0.001; Fig. 4A). The relative abundances of *Prevotella*, *Saccharofermentans*, *Stomatobaculum*, *Pseudobutyrivibrio*, and *Bacteroides* in the FG or MG groups were significantly higher than those in the EG group at 2 hours. The relative abundances of *Lawsonibacter*, *Sporobacter*, *Intestinimonas*, *Papillibacter*, *Ruminococcus*, *Butyricicoccus*, *Bacteroides*, and *Succiniclasticum* in the MG or FG groups were significantly higher than those in the EG group at both 8 hours and 24 hours (*P* < 0.05; Fig. 4B; Table S6 through S8).

### Comparison analysis of microbial predicted functional profiles

**Fig 5:**
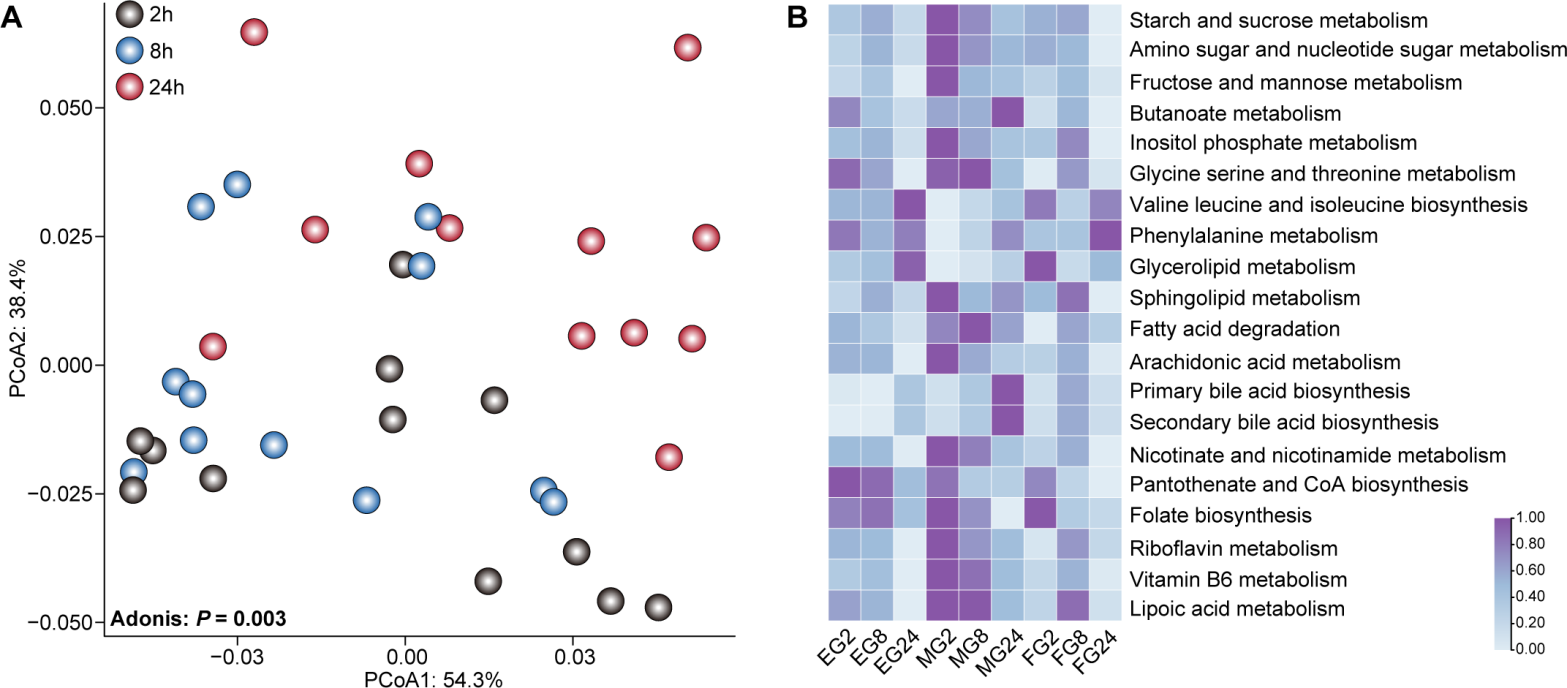
The predicted functional profiles with fermentation time *in vitro*. (**A**) PCoA results revealing variation of the predicted functions of rumen microbiota *in vitro* among the different fermentation times (2 hours = EG2, MG2, and FG2; 8 hours = EG8, MG8, and FG8; and 24 hours = EG24, MG24, and FG24) at Kyoto Encyclopedia of Genes and Genomes (KEGG) level 3 based on the Bray-Curtis dissimilarity. (**B**) Heatmap displaying significantly distinct pathways with fermentation in EG, MG, and FG groups at KEGG level 3. Color intensity indicates the normalized average relative abundances of each pathway, ranging from the minimum (light blue) to the maximum (purple).

We employed Tax4Fun to predict the potential functions and uncovered significant variations in metabolic pathways among the EG, MG, and FG groups (Bray-Curtis dissimilarity, *P* = 0.001; Fig. 5A), as well as across fermentation times within each group (Bray-Curtis dissimilarity, *P* = 0.003; Fig. S2A). Comparative analyses indicated that the relative abundances of pathways involved in starch and sucrose metabolism, amino sugar and nucleotide sugar metabolism, and fructose and mannose metabolism significantly decreased in the EG, MG, and FG groups as fermentation progressed (*P* < 0.05; Fig. 5B; Table S9 through S11). Further comparison revealed a total of 34, 18, and 34 significantly altered metabolic pathways among the EG, MG, and FG groups at 2 hours, 8 hours, and 24 hours, respectively (*P* < 0.05; Fig. S2B through D). For instance, at 2 hours, the MG group exhibited the significantly higher relative abundances in pathways related to starch and sucrose metabolism, amino sugar and nucleotide sugar metabolism, and fructose and mannose metabolism compared with the EG group (*P* < 0.05; Fig. S2B; Table S12). In contrast, at 2 hours, the FG group showed significantly higher relative abundances in pathways such as galactose metabolism; glycolysis/gluconeogenesis; cysteine and methionine metabolism; phenylalanine, tyrosine, and tryptophan biosynthesis; tyrosine metabolism; valine, leucine, and isoleucine biosynthesis; and glutathione metabolism compared with the EG group (*P* < 0.05). Additionally, at 8 hours, the MG group had significantly higher relative abundances of oxidative phosphorylation, nitrogen metabolism, and fatty acid degradation compared with the EG group. At 24 hours, the MG group also showed significantly higher relative abundances in amino sugar and nucleotide sugar metabolism; fructose and mannose metabolism; butanoate metabolism; citrate cycle, arginine, and proline metabolism; and alanine, aspartate, and glutamate metabolism compared with the EG group (*P* < 0.05; Fig. S2C and D ;Table S13 and S14).

### Co-occurrence network analysis of rumen microbiota and fermentation parameters

**Fig 6:**
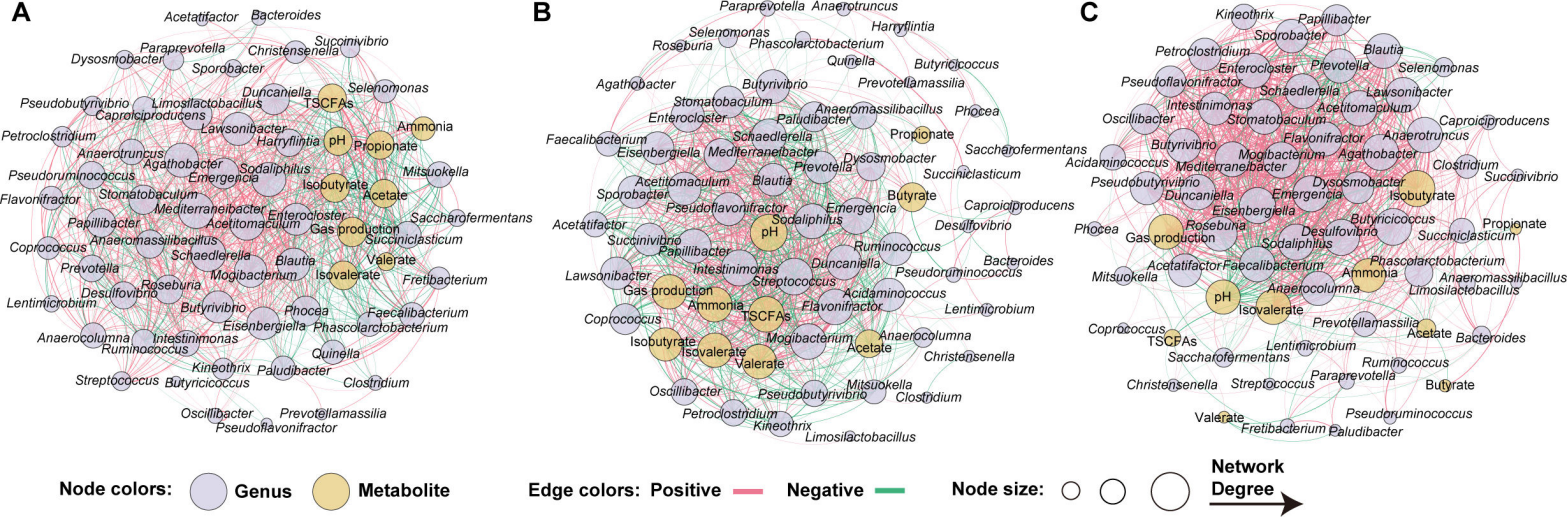
Co-occurrence network in rumen fermentation liquid *in vitro*. Network of the Spearman’s correlations among significantly different genera and fermentation parameters in EG (**A**), MG (**B**), and FG (**C**) groups.

We constructed a network to elucidate the correlations and co-occurrences between the significantly altered microbiota and fermentation parameters for the EG, MG, and FG groups, respectively (Fig. 6). In the EG group, the relative abundances of *Faecalibacterium*, *Selenomonas*, *Succiniclasticum*, *Clostridium*, and *Saccharofermentans* exhibited negative correlations with the concentrations of total SCFAs, ammonia nitrogen, and gas production (Fig. 6A). For the MG group, the relative abundances of *Butyrivibrio*, *Stomatobaculum*, *Flavonifractor*, and *Blautia* showed positive correlations with the concentrations of propionate, butyrate, or ammonia nitrogen (Fig. 6B). In the FG group, the relative abundances of *Roseburia*, *Pseudobutyrivibrio*, *Butyricicoccus*, *Butyrivibrio*, and *Clostridium* were positively correlated with the concentrations of ammonia nitrogen, isobutyrate, and isovalerate (Fig. 6C).

## DISCUSSION

In this study, we explored the dynamics of microbiota and metabolites in the rumen liquid of sika deer across three distinct stages of velvet antler growth using *in vitro* fermentation simulations. Our results indicated a rise in the production of total SCFAs, gas, and ammonia nitrogen from the EG stage to the FG stage, which is in line with our previous findings that total SCFAs and ammonia nitrogen are higher during the fast growth stage compared with the early stage (9). SCFAs, the primary metabolites resulting from rumen microbial fermentation, supply energy for the physiological functions of ruminants (17). Ammonia nitrogen in the rumen acts as a nitrogen source, supporting microbial growth and the synthesis of microbial proteins (18). Research has shown that dietary supplementation with arginine increases ammonia nitrogen concentrations in the rumen and enhances velvet antler production in sika deer (19). These findings suggest that the significant increase in fermentative activity within the rumen liquid may play a role in velvet antler growth.

We observed that the proportions of acetate and propionate were higher in the EG group than in the MG and FG groups, while the proportions of butyrate and branched-chain volatile fatty acids (valerate, isobutyrate, and isovalerate) were lower. This indicates a transition in rumen fermentation, with acetate and propionate dominating during the early stage of velvet antler growth, and butyrate becoming the dominant fermentation product during the metaphase and fast growth stages. This is consistent with our previous findings that the concentrations of butyrate and propionate increase with velvet antler growth (9), and the elevated levels of SCFAs in the rumen of sika deer were associated with improved velvet antler production (7). These results further highlighted the role of the shift in rumen fermentation pattern in velvet antler growth. It is known that fiber-rich and starch-rich diets promote the increased production of acetate and propionate in the rumen of cows, respectively (20). These results indicate increased carbohydrate metabolism by the rumen liquid microbiota during the early stage of velvet antler growth in sika deer. Acetate and propionate serve as energy sources or precursors for fatty acid synthesis and gluconeogenesis, while butyrate is metabolized by the rumen epithelium to produce acetoacetate and 3-hydroxybutyrate, providing energy for ruminants (13). Butyrate has gained increasing attention for its role in maintaining rumen epithelial homeostasis and integrity (21), inhibiting osteoclastogenesis and bone resorption, and influencing osteoblast precursors (22). It can also induce metabolic reprogramming in osteoclasts, leading to increased glycolysis and decreased oxidative phosphorylation, thereby inhibiting osteoclastogenesis (23). These results underscore the critical role of butyrate production in the later stages of velvet antler growth.

The predominance of phyla Bacteroidota and Bacillota in the rumen liquid is consistent with previous findings in the rumen contents of sika deer during velvet antler growth (9). The abundance of the phylum Bacillota increased over fermentation time in the MG and FG groups. It has been reported that members of the phylum Bacillota are major butyrate-producing groups (24), suggesting a significant contribution of Bacillota to butyrate production during velvet antler growth. Furthermore, the genera *Prevotella*, *Pseudoruminococcus*, and *Enterocloster* were dominated in the EG, MG, and FG groups. *Prevotella* is the most abundant bacterial genus during velvet antler growth (9); members of *Pseudoruminococcus* are capable of degrading complex carbohydrates (25). *Enterocloster* reclassified as a new genus within the Lachnospiraceae (26) plays a key role in protein degradation in the digestive tract of ruminants. These results highlight the key roles of *Prevotella*, *Pseudoruminococcus*, and *Enterocloster* in carbohydrate and protein metabolism of sika deer.

The proportion of *Stomatobaculum* and *Blautia* in the three groups increased over fermentation time. *Blautia* can ferment oligosaccharides to produce propionate through the propylene glycol pathway (27). Our previous finding showed that *Stomatobaculum*, a SCFA-producing bacterium, increased significantly in rumen liquid during antler growth (9). These results suggest that the increased abundances of *Stomatobaculum* and *Blautia* contributed to the rise in SCFA level over fermentation time. Additionally, the proportion of *Sporobacter* and *Butyricicoccus* in the FG group increased over fermentation time. Members of *Sporobacter* are involved in the uptake and metabolism of SCFAs (28), while *Butyricicoccus* has been shown to produce butyrate through the fermentative breakdown of recalcitrant plant fibers (29). The increased presence of these microbes may lead to higher butyrate production in the FG group. The proportions of *Prevotella*, *Saccharofermentans*, and *Bacteroides* were higher in the MG or FG groups compared with the EG group at 2 hours, coinciding with increased metabolic pathways related to starch and sucrose metabolism and galactose metabolism. Moreover, the proportions of *Lawsonibacter*, *Sporobacter*, *Papillibacter*, *Butyricicoccus*, *Bacteroides*, and *Succiniclasticum* were higher in the MG or FG groups compared with the EG group at 24 hours. *Prevotella*, *Saccharofermentans*, and *Bacteroides* exhibit the fiber degradation activity (30, 31), and *Bacteroides* possess various polysaccharide utilization loci that facilitate efficient and synergistic degradation of complex polysaccharides (32). *Succinatimonas* ferments glucose and other carbohydrates to produce large amounts of acetate and succinate, with the succinate being further oxidized to produce butyrate (33). *Lawsonibacter* and *Papillibacter* are generally recognized as the primary producers of butyrate (34, 35). Similarly, the predicted functional analysis of metabolic pathways, including fructose and mannose metabolism, butanoate metabolism, and citrate cycle, also showed higher activity in the MG group compared with the EG group. Our previous study revealed that the increased production of velvet antler in sika deer is accompanied by enhanced butyrate metabolism and the citrate cycle of the gut microbiota (36). The microbial metabolite butyrate has been demonstrated to regulate bone development by activating the Wnt pathway in osteoblasts (10). The citrate cycle provides chondrocytes with a plethora of ATP, thereby facilitating their replication, differentiation, and the synthesis of an extracellular matrix (37). These findings suggest the capability of rumen microbiota to generate butyrate during the metaphase and fast growth of velvet antler.

Our findings also revealed significant differences in the relationships between the microbiota and fermentation parameters across the three groups. A notable negative correlation was identified between the proportions of *Clostridium* and the concentration of ammonia nitrogen in the EG group. Previous studies have indicated that *Clostridium* members primarily exhibit proteolytic activity within the rumen (38), suggesting that *Clostridium* likely plays a role in protein metabolism in the rumen at the early stage of velvet antler growth. In the MG group, positive correlations were observed between the concentration of butyrate and the proportion of *Stomatobaculum*, *Butyrivibrio*, and *Blautia*, which are metabolically active bacteria known for producing butyrate as a key metabolite (29, 39, 40). In the FG group, positive correlations were noted between the concentration of isobutyrate and isovalerate and the proportions of *Roseburia*, *Pseudobutyrivibrio*, *Butyricicoccus*, *Butyrivibrio*, and *Clostridium*. Isobutyrate and isovalerate are products of branched-chain amino acid metabolism, and *Roseburia* and *Clostridium* have been implicated in the metabolism of valine, leucine, and isoleucine (41, 42). Wang et al. (43) found that the population of *Butyribrio* increased in the rumen of cattle when their diet was supplemented with isobutyrate (43). These results suggest that changes in rumen fermentation are likely inducted by the specific rumen microbiota during velvet antler growth. However, our results are based on a limited sample size. It is well established that rumen microbiota exhibit redundancy and resilience (44). Therefore, the application of metagenomics and transcriptomics is essential to clarify the metabolic shifts within the rumen microbiome during velvet antler growth, particularly with a larger sample size.

### Conclusions

In the present study, we characterized the dynamics of microbiota and metabolites using *in vitro* rumen fermentation of sika deer during velvet antler growth. Our findings revealed an increased production of SCFAs and ammonia nitrogen across the EG, MG, and FG groups as fermentation progressed. Moreover, we detected a transition in rumen fermentation patterns from an acetate- and propionate-dominated profile during the early stages to a butyrate-dominated profile in the later stages of velvet antler growth. The distinct microbiota profiles observed at different fermentation times underscored the shifts in microbial communities throughout the velvet antler development process.

## MATERIALS AND METHODS

### Animals and sample collection

A total of 15 healthy, adult male sika deer, aged 4 years, were utilized from our previous studies (9). Briefly, the deer were categorized into three groups based on the stages of velvet antler growth: deer with velvet antlers casting stage (0 day, early antler growth, EG group), deer with velvet antlers growing for 30 days since casting (metaphase antler growth, MG group), and deer with velvet antlers growing for 45 days after the casting (fast antler growth, FG group). The animals were slaughtered 3 hours after morning feeding, and rumen content was collected, homogenized, mixed, and filtered through four layers of cheesecloth to obtain rumen liquid (approximately 2 L). The rumen liquid from each group was thoroughly mixed to prepare the rumen inoculum for the *in vitro* experiment. All experimental animals were handled in accordance with the guidelines for animal research of the Animal Ethics Committee of Jilin Agricultural University (Approval No: 20210314002).

### *In vitro* ruminal liquid fermentation

The gas production system (RFS, ANKOM, USA) was employed for *in vitro* fermentation as per established methods (45). Each culture bottle was filled with a premixed 120 mL solution (80 mL buffer and 40 mL rumen liquid), flushed with CO_2_ for about 30 s, immediately capped, and placed in an air bath incubator (HNY-211B, Honour, Tianjin) for cultivation. The GPM (ANKOM, USA) software was utilized to monitor and record gas production. Whole corn silage, a common forage in the sika deer production system, was used as a substrate (dry matter (DM) = 34%, starch = 29.13% DM, crude protein (CP) = 7.29% DM, neutral detergent fiber (NDF) = 42.03% DM, and acid detergent fiber (ADF) = 22.26% DM). Culture bottles were removed and placed in ice water to halt fermentation at 2 hours (EG2, MG2, and FG2 groups), 4 hours (EG4, MG4, and FG4 groups), 6 hours (EG6, MG6, and FG6 groups), 8 hours (EG8, MG8, and FG8 groups), 12 hours (EG12, MG12, and FG12 groups), and 24 hours (EG24, MG24, and FG24 groups) of *in vitro* incubation. Each time point had four replicates (one bottle per replicate). The rumen fermentation liquid was collected, immediately frozen in liquid nitrogen, and stored at −80°C for subsequent analysis.

### Measurement of fermentation parameters

The pH of rumen fermentation liquid was measured by a pH meter (MP511, Sanxin, Shanghai). The concentration of SCFAs in rumen fermentation liquid was determined using a gas chromatograph (7890B, Agilent, UK), following a previously established method (9). Gas production was quantified using the ANKOM RFS gas production system, with pressure in each culture bottle directly transmitted to a computer for detection via a sensor. The concentration of ammonia nitrogen was determined by colorimetric method described by Chaney and Marbach (46).

### DNA extraction, 16S rRNA gene, sequencing, and bioinformatic analysis

Total genomic DNA of fermentation liquid were extracted using the beat-beating method (47). The V1–V9 region of the 16S rRNA genes was amplified with the primer pair 27F (5′-AGRGTTYGATYMTGGCTCAG-3′) and 1492R (5′-RGYTACCTTGTTACGACTT-3′) (48). The resultant amplicons were purified using the AxyPrep DNA Gel Extraction Kit (Axygen Biosciences, CA, USA). SMRTbell libraries were prepared from the amplified DNA by blunt-ligation, which were then sequenced on the PacBio Sequel platform. The sequences were used to cluster OTUs with 98.65% similarity by UPARSE (49), and UCHIME (50) was employed and remove chimeric sequences. Representative sequences of each OTU were analyzed with the RDP Classifier against the SILVA (SSU138) database, with a confidence threshold of 0.7 (51). The microeco package was used to calculate the alpha diversity indices, including the Shannon and Simpson indices (52). The PCoA based on the Unweighted UniFrac distance was used to analyze the difference of microbial community. Permutational analysis of multivariate dispersions was used to assess sample dispersion within each group by calculating distances from the group centroid. Differences were determined by the permutational multivariate analysis of variance (PERMANOVA), with *P* values based on 999 permutations. Tax4Fun was used to predict the functional profiles summarized at the Kyoto Encyclopedia of Genes and Genomes (KEGG) pathway level (53). Spearman’s correlation coefficient between significantly changed microbiota and fermentation parameters in EG, MG, and FG groups was calculated using the Hmisc package, respectively (54). The significant correlations (*P* ≤ 0.05, |rho| > 0.5) were visualized using Gephi (55).

### Statistical analysis

The Kruskal-Wallis (KW) test was employed to determine differences in the concentration of SCFAs and ammonia nitrogen, pH, and alpha diversity indices, as well as the relative abundances of taxonomy and KEGG pathways. The false discovery rate of the Benjamini-Hochberg method was applied, with a threshold of *P* < 0.05 considered statistically significant. When the KW comparison was significant, the Wilcoxon rank sum test was used to determine the significance of each comparison.

## Data Availability

Raw sequence reads for all samples are available under NCBI project PRJNA1110357.

## SUPPLEMENTAL MATERIAL

Supplemental figures spectrum.02829-24-s0001.docx Fig. S1 and S2.

Supplemental tables spectrum.02829-24-s0002.xlsx Tables S1 to S14.

ASM does not own the copyrights to Supplemental Material that may be linked to, or accessed through, an article. The authors have granted ASM a non-exclusive, world-wide license to publish the Supplemental Material files. Please contact the corresponding author directly for reuse.
