# Supplementary material for: In vitro dynamics of rumen microbiota and fermentation profiles with Antler growth of Sika deer
Source: Microbiol Spectr. 2025 Jan 28;13(3):e02829-24. doi: 10.1128/spectrum.02829-24 (PMC11878070; doi:10.1128/spectrum.02829-24)
Supplement: Supplemental figures — Fig. S1 and S2. [file spectrum.02829-24-s0001.docx]

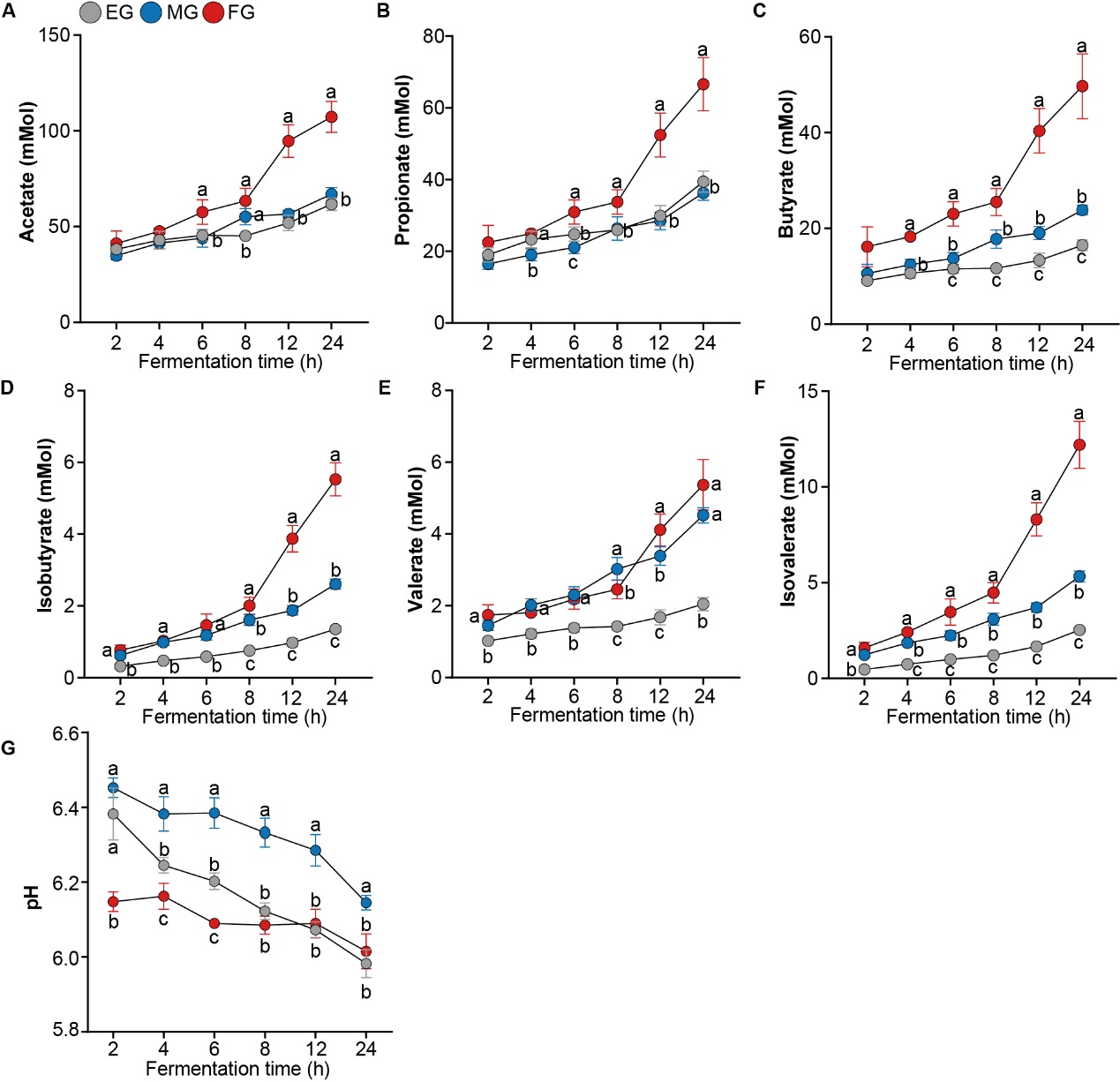


**Figure S1.** Comparison of the concentrations of acetate (**A**), propionate (**B**), butyrate (**C**), isobutyrate (**D**), valerate (**E**) and isovalerate (**F**), and the pH (**G**) in rumen fermentation liquid among EG, MG and FG group with fermentation. a, b and c indicate *P* < 0.05 among three groups at same time.


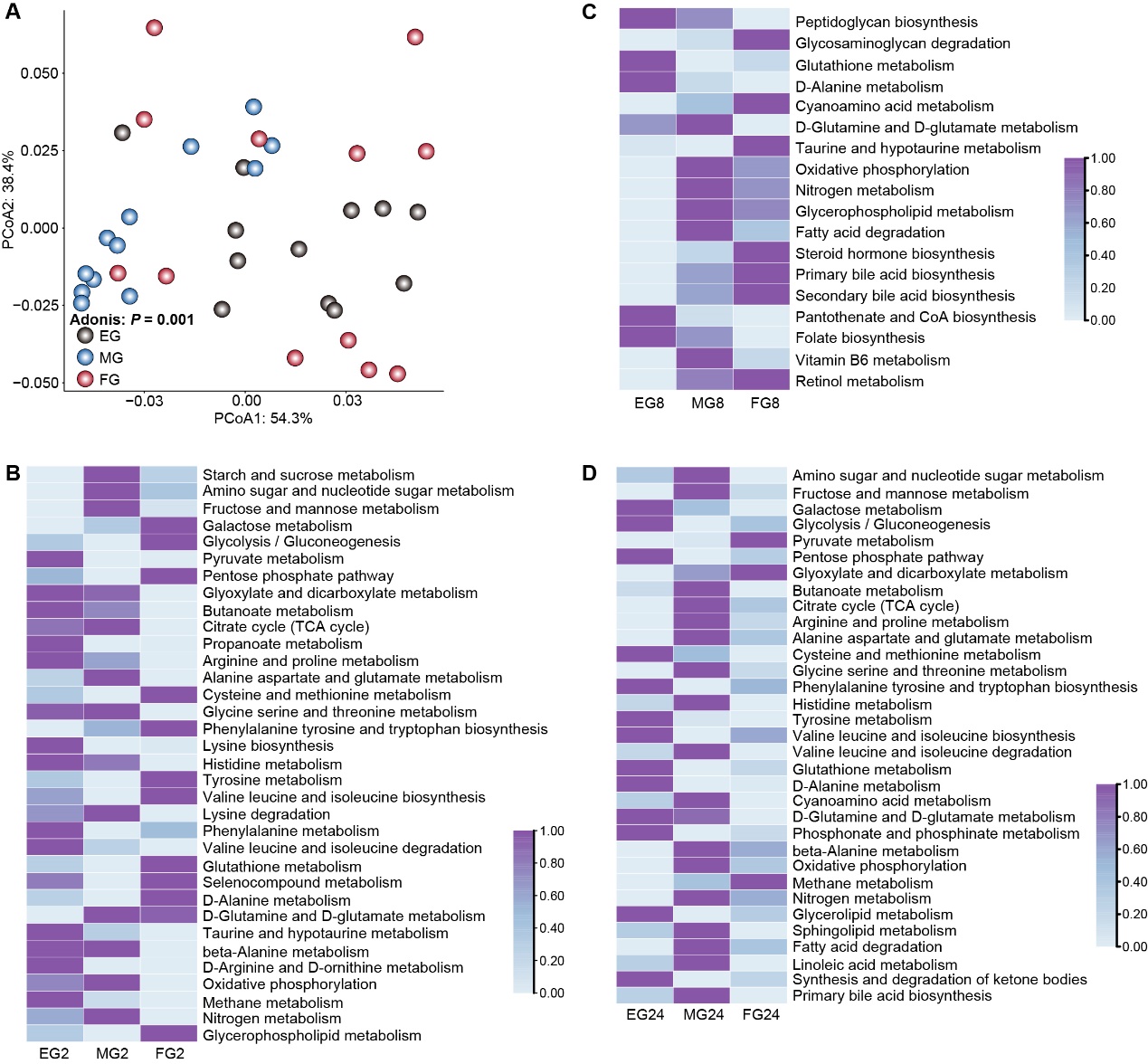


**Figure S2.** PCoA results revealing the variation of the predicted functions of rumen microbiota *in vitro* during antler growth at KEGG level 3 based on the Bray-Curtis dissimilarity (**A**). Heatmap showing the significantly distinct pathways at 2 h (**B**), 8 h (**C**), 24 h (**D**) in EG, MG and FG groups at KEGG level 3. Rectangle colors indicate the normalized average relative abundances of each pathway from the minimum (pool blue) to maximum (purple).
